# Margin-Based Pareto Ensemble Pruning: An Ensemble Pruning Algorithm That Learns to Search Optimized Ensembles

**DOI:** 10.1155/2019/7560872

**Published:** 2019-06-03

**Authors:** Ruihan Hu, Songbin Zhou, Yisen Liu, Zhiri Tang

**Affiliations:** ^1^Guangdong Key Laboratory of Modern Control Technology, Guangdong Institute of Intelligent Manufacturing, Guangdong Academy of Sciences, Guangzhou 510070, Guangdong Province, China; ^2^School of Physics and Technology, Wuhan University, Wuhan 430072, Hubei, China

## Abstract

The ensemble pruning system is an effective machine learning framework that combines several learners as experts to classify a test set. Generally, ensemble pruning systems aim to define a region of competence based on the validation set to select the most competent ensembles from the ensemble pool with respect to the test set. However, the size of the ensemble pool is usually fixed, and the performance of an ensemble pool heavily depends on the definition of the region of competence. In this paper, a dynamic pruning framework called margin-based Pareto ensemble pruning is proposed for ensemble pruning systems. The framework explores the optimized ensemble pool size during the overproduction stage and finetunes the experts during the pruning stage. The Pareto optimization algorithm is used to explore the size of the overproduction ensemble pool that can result in better performance. Considering the information entropy of the learners in the indecision region, the marginal criterion for each learner in the ensemble pool is calculated using margin criterion pruning, which prunes the experts with respect to the test set. The effectiveness of the proposed method for classification tasks is assessed using datasets. The results show that margin-based Pareto ensemble pruning can achieve smaller ensemble sizes and better classification performance in most datasets when compared with state-of-the-art models.

## 1. Introduction

Recent publications have widely applied multiple classifier systems (MCSs) [1] in fields such as digital recognition [2], facial recognition [3], acoustic recognition [4], credit scoring [5], imbalance classification [6], recommender system [7], software bug detection [8], and environmental data analysis [9]. Unlike deep learning frameworks [10], it has been shown that MCSs [2] can be learned well on both small and large-scale sets. The advantage of an MCS is that more decision guidelines are provided by the ensemble pool than by a single learner. However, MCSs cannot determine which learners are most suitable with respect to the incoming dataset because not all decision guidelines are useful for classifying targets.

As a modified case of MCS, an ensemble pruning system (EPS) [11–20] is a popular machine learning model that can select base learners from an ensemble pool to construct the expert. Previous studies [12] have demonstrated that EPS can achieve superior performance to MCS because the competence level of each base learner is calculated using the validation set, and learners with low competitiveness that unlikely improve the performance of ensemble pool are pruned when the testing set is used in EPS. In general, the EPS model can be separated into 3 categories: static pruning, dynamic classifier pruning, and dynamic ensemble pruning. In static pruning [13], experts are directly selected from the ensemble pool using the training set. In dynamic classifier pruning [14, 15], only the most competent learner can be chosen from the ensemble pool once a test sample emerges. In dynamic ensemble pruning [16–20], the subsets of the ensemble pools are selected as experts to deal with the test samples. For our viewpoint, the dynamic classifier pruning model can be seen as a special case of the dynamic ensemble pruning model. The dynamic ensemble pruning model has four stages: overproduction, region of confidence definition, selection, and integration. During overproduction, every base learner independently learns when composing the ensemble pool using the training set. During region of competence definition, the criterion is defined to explore the correlation between the ensemble pool and the targets using the validation set. During selection, according to the region of competence, different learners with low competitiveness need to be pruned from the testing set. During integration, the outputs of the learners are aggregated. To the best of our knowledge, different EPS models use different definitions of the region of competence criteria to select classifiers. Antosik and Kurzynski [16] estimated the source competence of every learner using the minimum difference minimization criterion between the outputs and the targets with respect to the validation set. Lobato et al. [17] used the Bayesian computation framework to approximate the confidence level of the remaining learners by querying the selected expert. The size of the expert is increased until the confidence level of the remaining learners is above the predefined level. Li and Zhou [18] generalized the EPS problem as a quadratic programming problem with a sparse solution. The results in this publication show that dynamic ensemble pruning can achieve good generalization performance little expertise. KNORA-Eliminate [19] selected the expert that can correctly classify all samples in the region of competence. The region of competence for KNORA-Eliminate will be reduced until the expert achieves 100% accuracy in the region of competence. META-DES [20] provided an alternative framework that views dynamic ensemble pruning as a meta-learning procedure. The meta-features are constructed according to the correlation among the probability outputs, inputs, and targets.

Regarding dynamic ensemble pruning, the size of the ensemble pool has been fixed in previous publications [16–20], and these dynamic ensemble pruning methods failed to search the optimal ensemble pool during the overproduction stage. According to Davis et al. [21], the selection of the expert is known to be a nondeterministic polynomial hard (NP-hard) problem. Additionally, most pruning criteria estimate the competency of the learners but neglect the condition that queries the sample located in the indecision boundary, and learners have different suggestions for samples in the region of competence. It is insufficient to correctly select the expert in the ensemble pool. To address these problems, we first regard the calculation procedure for the size of the pool during the overproduction stage as an optimization problem. Two targets, i.e., ensemble pool size and learning performance for dynamic ensemble pruning models, are globally searched by a bi-objective programming formulation. The ensemble pool size calculated by the programming method does not utilize the local information about the region of competence for the validation set. Thus, the obtained ensemble pool size may be suboptimal. More precisely, the local exploration method between base learners in the ensemble pool with the suboptimal size needs to be considered. Accordingly, margin-based Pareto ensemble pruning (MBPEP) is proposed, consisting of Pareto optimization and a margin criterion pruning mechanism for global and local search of the optimal ensemble pool size. In this paper, the following two contributions are made: the Pareto optimization algorithm [22] is applied to calculate the optimal size of the ensemble pool during the overproduction stage for dynamic ensemble pruning in MBPEP, and the margin criterion pruning (MCP) mechanism is contained within the MBPEP so that the learners in the indecision region can detect the different classes in the region. Subsequently, the margin criterion for each class in the indecision region is further calculated to prune the size of the expert. Classifiers in the indecision region that contain a lot of information will be pruned according to the MCP. Unlike some dynamic ensemble pruning methods [19, 20] based on various pruning criteria, the local exploration technique in MBPEP is preselected for these pruning criteria; it can be directly inserted into them and provide more robust learning performance than the original dynamic ensemble pruning methods.

The remainder of this paper is organized as follows.  Section 2.1 describes the proposed MBPEP framework.  Section 2.2 discusses the Pareto optimization that estimates the optimal size of the ensemble pool.  Section 2.2 introduces the MCP method to prune the ensemble pool. In Section 3, a series of experiments are executed to evaluate the performance of the MBPEP framework. The study's conclusions are given in Section 4.

## 2. Methodology

In this section, the methodology of the MBPEP framework is discussed, and the basic architecture of MBPEP is described in Section 2.1. Key ensemble exploration details for ensemble pool, Pareto optimization, and MCP are described in Sections 2.2 and 2.3.

### 2.1. Margin-Based Pareto Ensemble Pruning (MBPEP) Framework for Dynamic Ensemble Pruning

Like the general dynamic ensemble models [16–20], the MBPEP-based dynamic ensemble pruning framework can be separated into four stages. During the overproduction stage, *T* is the initial ensemble pool size, and each learner in ensemble pool **H**={*H*_1_,…, *H*_*T*_} can be independently learned based on the training set *X*_Tr_. To keep the ensemble pool diverse and informative during this stage, these *T* base learners will be initialized by training different training sets. For example, bagging, boost, and clustering approaches [1] that can generate different distributions of training sets are used to train every learner.

**Figure 1:**
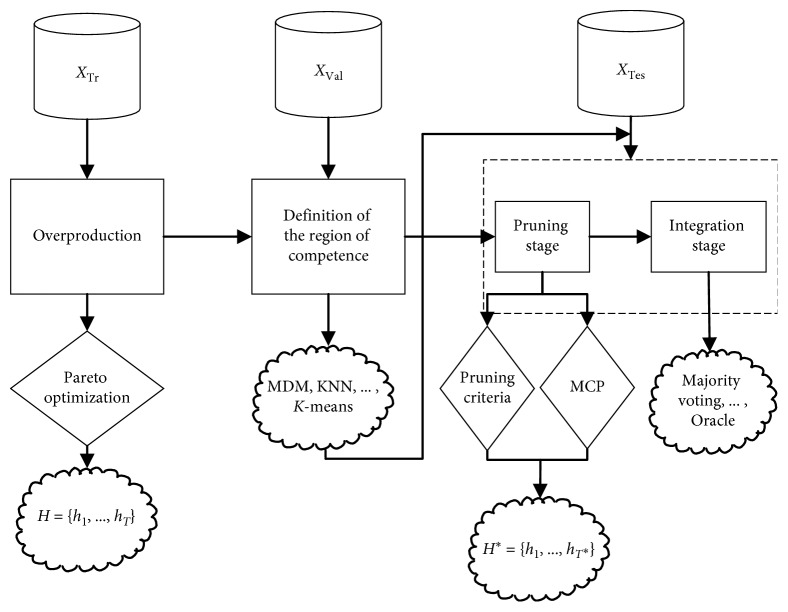
Architecture of MBPEP, which consists of four stages: overproduction, region of competence definition, pruning, and integration.

Bagging is used in this paper, and classification and regression trees (CARTs) [23] are used as base learners. Subsequently, the optimal overproduction pool size **H**_**S**_={*H*_1_,…, *H*_*T*_s__} is estimated using the Pareto optimization algorithm. The global exploration for the *T* initial base learners is calculated, and the *T*_s_ learners that result in suboptimal performance are selected during this stage. During the region of competence definition stage, the neighbors in validation set *X*_Val_ are learned using various criteria. Based on region of competence definition, similar samples for unknown query instance *X*_query_ and the competence level of each base learner can be estimated. The common techniques for defining the region of competence include minimum difference minimization [16], *k*-nearest neighbors (KNN) [24], *K*-means [25], and the competence map method [26]. During the pruning stage, some learners are extracted to construct the expert with respect to the test set *X*_Tes_. In contract, traditional dynamic ensemble pruning models use insufficient pruning techniques such as KNORA-Eliminate [19] and META [20] to prune learners with low competitiveness in the ensemble pool. In MBPEP, the expert **H**^*∗*^={*H*_1_,…, *H*_*T*^*∗*^_} is estimated through two steps for the test set. Meanwhile, the region of competence for *X*_Tes_ will be checked using MBPEP with respect to whether the learners in the ensembles have the same suggestions when they recognize the samples in the region of competence for *X*_Tes_. When every learner in the ensembles has the same suggestion, that region is defined as a safe region; in this condition, the MCP method does not need to be activated, and the ensemble pool is passed down to various pruning techniques. However, when the learners in ensembles make different suggestions for samples in the region of competence for *X*_Tes_, the region is defined as the indecision region. MCP is applicable to the scope of the indecision region, and the size of the expert can be further pruned. The MCP operator, which does not damage the general pruning criteria [19, 20], can be used as a preselector to improve exploration for the local information between the base learners in the ensemble pool. During the integration stage, the results that are calculated by the expert are aggregated. The strategies used for aggregation can be separated into three types: nontrainable methods [1], in which the weights of the outputs do not need to be learned; trainable methods [1], in which the outputs from the experts in the pruning stage are used as input features to be trained by another learner; and dynamic weighting methods [1], in which the weights are determined by the estimated competence levels of every expert. The famous integration methods include majority voting and oracle. In this paper, majority voting is used as the aggregation strategy. The architecture of the MBPEP-based dynamic ensemble pruning framework is shown in Figure 1. Notably, the performance of dynamic ensemble pruning is sensitive to the definition of the region of competence. Since the region of competence is not the key contribution of MBPEP, in this paper, different criteria are used to define the region of competence.

### 2.2. Pareto Optimization Evolutionary Algorithm

In recent years, several studies [11–20] have demonstrated that ensemble models always achieve better results than any base learner. However, methods of building ensembles that can achieve great performance with respect to the test set are still required. In this paper, this problem is solved by MBPEP during the overproduction stage. In Section 2.1, the architecture of MBPEP that is introduced during the overproduction stage in Figure 1 is discussed, and the overproduced learners are trained to compose the ensemble pool. In general, it is a conflicting phenomenon that few base learners produce few decision boundaries without achieving powerful performance. According to previous studies [16–20], the ensemble pool size is fixed during the overproduction stage, and calculation of the size of the ensemble pool that is suitable for handling classification tasks is challenging. Our objective is to estimate an optimal ensemble pool size for achieving better performance than that of a fixed ensemble pool size. The MBPEP computation framework uses Pareto optimization to address the learning performance and the ensemble pool size.

In this study, the initial ensemble pool size is *T* (**H**={*H*_1_,…, *H*_*T*_}), the inputs are **X**={*X*_1_,…, *X*_*n*_}, and the targets are **y**={*y*_1_,…, *y*_*n*_}, where *n* is the number of instances and *c* is the number of classes. The corresponding optimized pool size after calculation is defined as **H**_**S**_. The vector representation form of the optimized ensemble pool is composed of a binary element vector that is defined as *S* ∈ {0,1}^*T*^ with *T* dimensions. *S*_*i*_=1 denotes that the *i*_th_ learner in **H**={*H*_1_,…, *H*_*T*_} is selected; otherwise, *S*_*i*_=0. The Pareto optimization algorithm converts the calculation of the optimal ensemble pool size into a subset selection problem. Suppose that the outputs of the different ensemble pools can be denoted as *h*_*i*,*s*,*k*_ (*i* ∈ 1,…, *n*, *s* ∈ [0, *T*] and *k* ∈ 1,…, *c*), where *i*, *s*, and *k* denote the indexes of instances, ensembles, and classes, respectively. Following these symbols, the classification error *f*(**H**_**S**_) can be denoted as follows:

(1) $$\begin{array}{c} f\left(H_{S}\right)=1-\frac{1}{n}\overset{n}{\underset{i=1}{\sum }}\overset{c}{\underset{k=1}{\max}}\overset{T}{\underset{s=1}{\sum }}h_{i,s,k} \end{array}$$

The classification performance and ensemble pool size are estimated using the Pareto optimization bi-objective programming formulation, as follows:

(2) $$\begin{array}{c} \underset{S\in {\left\{0,1\right\}}^{T}}{\arg\min}\left(f\left(H_{S}\right),L\right), \end{array}$$

where *L* denotes sum(**S**). The operator sum(·) denotes that the binary vector **S** is summed up to calculate the ensemble pool size. Unlike other single-objective algorithms [27, 28], the concept of “domination,” which continuously disturbs the ensemble size and measures the difference between *f*(**H**_**S**_) and *L* during the global searching iteration process, is introduced in Pareto optimization.

### 2.3. Margin Criterion Pruning (MCP)

**Figure 2:**
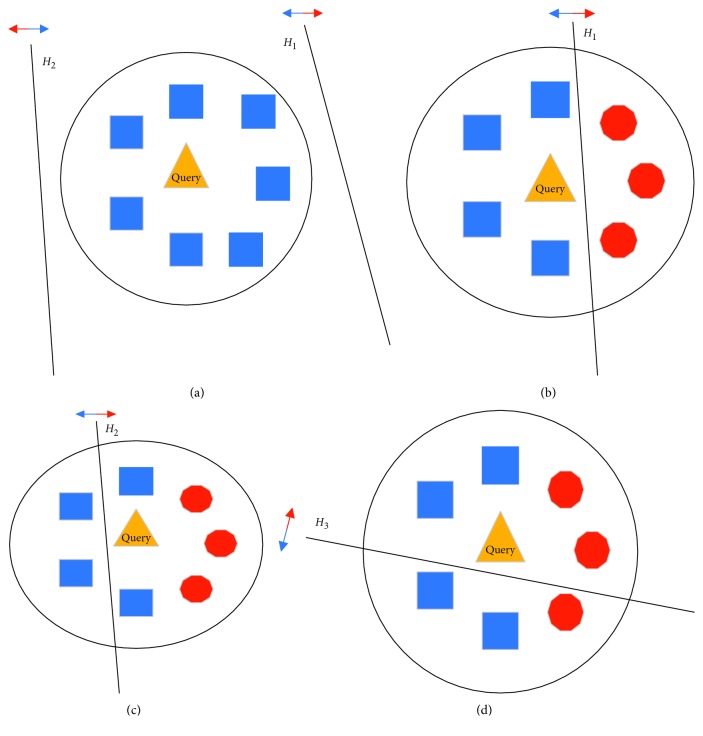
The region of competence during the pruning stage. The query sample is located in (a, b) the safe region and (c, d) the indecision region.

As mentioned in Section 2.1, the Pareto optimization method is used during the overproduction stage to calculate the optimal ensemble pool size in the dynamic ensemble pruning model. If query sample *x*_query_ is located in the indecision boundaries of the learners, then the predictions of the learners for query sample *x*_query_ might differ from the samples that belong to its corresponding region of competence. Like the other characteristics of heuristic algorithms [22], the global exploration between the base learners used by Pareto optimization with respect to the two objectives (*f*(**H**_**S**_) and sum(**s**)) cannot deal with this condition. Pareto optimization provides the suboptimal [21] ensemble pool size during the overproduction stage because the local information between the base learners and the targets is neglected in the solution space. Different from other publications, previous studies [16–20] propose using the pruning criteria to estimate the competence of each learner for query sample *x*_query_ without considering when this query sample is located in the indecision boundaries. In this paper, the local information is measured by MCP mechanism between these base learners in the ensemble pool to compute the information quantity that query sample *x*_query_ is located in the indecision boundaries of the learners. The MCP is added during the pruning stage as a preselector that is executed in parallel with such classical pruning criteria [16–20]. As a subset of the region of competence, the indecision region of the ensemble pool is defined and explored to estimate the local competence of each sublearner in the ensembles. A schematic diagram of the region of competence for four ensemble pool size conditions is shown in Figure 2.

The safe region and the indecision region are defined and shown in Figures 2(a) and 2(b) and Figures 2(c) and 2(d), respectively. As shown in Figure 2, two regions of competence that query samples (yellow triangle in Figure 2) located in the safe and indecision regions are determined according to whether the ensembles can correctly recognize the neighbors of the query sample. If these ensembles make the same suggestions for these neighbors, the region of competence becomes the safe region. When ensembles have different suggestions for these neighbors, the region of competence becomes the indecision region. According to Figures 2(b)–2(d), it can be seen that ensembles can reach 100% (7/7), 71.4% (5/7), and 57.1% (4/7), respectively. In addition, MCP focuses on the indecision region, and the local competence of each learner that can distinguish the samples with different classes in the indecision region is estimated.

Suppose that there are *T*_S_ learners **H**_**S**_={*H*_1_,…, *H*_s_} (in Section 2.2) selected to compose the ensemble pool after being globally explored using the Pareto optimization algorithm during the overproduction stage. Suppose that query sample *x* with *N* neighbors in the region of competence has various suggestions made by the ensembles. *N*_*x*_^1^ denotes the number votes of the most popular label voted for by the ensembles **H**_**S**_. In addition, *N*_*x*_^2^ represents the number votes of the second most popular label voted for by *H*^*∗*^. The marginal criterion is used to measure the amount of information for the ensembles in the indecision region using MCP. We define the difference between the labels with the most and second most votes as the marginal information for the neighbors of query sample *x* as follows:

(3) $$\begin{array}{c} \text{margin}\left(x\right)=\frac{N_{x}^{1}-N_{x}^{2}}{T_{\text{S}}}. \end{array}$$

Entropy is used to compute the amount of information quantity in the machine learning field [29]. To measure the margins of the final hypotheses, the margin entropy criterion is defined to calculate the amount of information of samples *X* for *H*_*i*_ in **H**_**S**_={*H*_1_,…, *H*_s_}:

(4) $$\begin{array}{c} C_{H_{i}}\left(X\right)=-\frac{1}{N}\overset{N}{\underset{j=1}{\sum }}\log\left[\text{margin}\left(x_{j}\right)\right], \end{array}$$

where *N* denotes the number of neighbors around query samples *X*. If a greater difference is calculated by equation (3) between the most and second most popular labels, then it can be interfered that these ensembles cannot detect samples that belong to different classes in the region of competence. In addition, in MBPEP, *N*_*x*_^1^ − *N*_*x*_^2^ is always less than *T*_S_, and hence, the maximum value of margin(*x*) is less than 1. According to equation (4), it can be seen that, if the calculated margin entropy *C*_*H*_*i*__(*X*) of validation sample *X* is more than 0, then the learner can be used to compose the expert. Otherwise, when *C*_*H*_*i*__(*X*) ≤ 0, the learner needs to be pruned. This process is called MCP. MCP performs greedy research to define a subset of the ensemble pool that can be used as a preselector to determine a suitable learner that can recognize the different targets in the indecision region. The MCP algorithm is shown in Algorithm 1.

## 3. Results and Discussion

### 3.1. Configuration

Sixteen UCI datasets [30] are used as benchmarks in this paper. The characteristics of these datasets are shown in Table 1, and each of them is split into three parts: a training set, a validation set, and a test set. Classification and regression trees (CARTs) are used as base learners. First, the bagging method is applied to split the training datasets. The training sets are divided into subsets, and each subset is selected using bootstrap extraction. The subclassifiers are trained using the training set. The number of ensembles is preset as 100. Second, the MBPEP method is applied to prune the validation set. The optimized subclassifiers are aggregated into the final hypotheses using the majority voting method [1]. Finally, the test set is used to measure the performance of the methods.

### 3.2. Pruning Metrics

The bagging ensemble is a common architecture that uses learners to train different bootstrapped samples. In this paper, the full bagging method in which all base learners are selected to construct the ensemble classifiers is used as the baseline algorithm. The classification accuracy and ensemble size of MBPEP are compared with the optimization-based pruning method EA [31] and four competence ordering-based pruning methods, i.e., reduced error pruning [32], kappa pruning [33], complementarity measure pruning [34], and margin distance minimization pruning [26]. These pruning methods are described as follows:

- Optimization-based pruning [31] regards aggregation as a programming problem that intends to select the optimized subset of base learners by minimizing the validation error. In this paper, EA is used as an optimization ensemble method.
- Reduced error (RE) pruning [32] sorts the subclassifiers and adds these subclassifiers one by one to find the lowest classification error of the final ensemble. Margineantu and Dietterich use the back fitting search method to approximate the generalization performance of RE pruning.
- Kappa pruning [33] uses the Kappa error as a statistical method that measures the diversity between a pair of sublearners. Kuncheva uses *κ* statistics to measure the correlation between the outputs of two sublearners. The classifiers are iteratively added to the ensemble with the lowest *κ* statistics.
- Complementarity measure (CM) pruning [34] is an ordered pruning ensemble that focuses on finding the most complementary learners to the ensemble for each iteration and incorporates them into the ensemble classifier. The complementarity pruning method enhances the performance of the classes with the most votes without harming the classes with the least votes.
- Margin distance minimization (MDM) pruning [16] was discussed in Introduction.
- Randomized reference classifier (RRC) pruning [26] estimates the competence of each learner in the ensemble pool for the validation set using the corresponding probability outputs and several random variables with beta probability distribution.
- KNORA-Eliminate [19] was discussed in Introduction.
- META-DES [20] was discussed in Introduction.

### 3.3. Characteristics of MBPEP

To investigate the characteristics of MBPEP, the advantages of both Pareto optimization and MCP are measured. In this section, RRC, KNORA-Eliminate, full bagging, and META-DES are used for comparison purposes. Notably, the full bagging method can be used as the baseline without pruning. The two contributions (Pareto optimization and MCP) for MBPEP are applied to the overproduction and pruning stages, respectively, and hence, two experiments in which MCP and MBPEP are embedded into dynamic ensemble pruning models which are executed to explore the characteristics of MBPEP.

**Figure 3:**
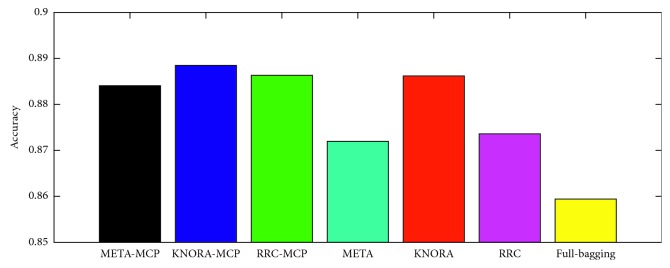
Performance of the dynamic ensemble pruning methods with and without MCP.

**Figure 4:**
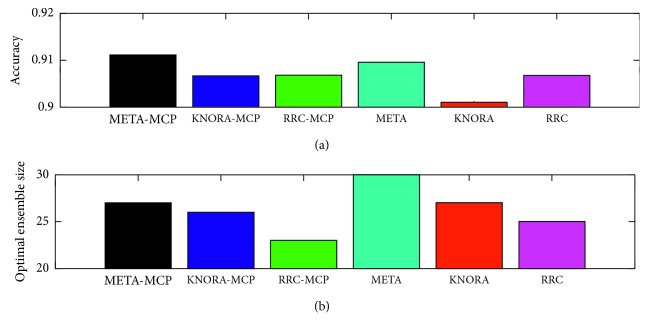
Performance of the dynamic ensemble pruning models with and without MBPEP.

To measure the advantages of MCP, from Figure 3, we can see that the performances of the dynamic ensemble pruning models (for the META and KNORA models) based on MCP are superior to those without MCP. In addition, all EPS models achieve better performance than full bagging. As an alternative technique for measuring performance, the *F*1 value is an effective metric that has been widely [35] used to measure the precision and sensitivity of results, and it is calculated as follows:

(5) $$\begin{array}{c} F1=\frac{2\text{TP}}{2\text{TP}+\text{FP}+\text{FN}}, \end{array}$$

where TP, FP, and FN denote true positives, false positives, and false negatives, respectively. For example, for class 0 in Figure 3, the *F*1 values for META-DES, KNORA-Eliminate, and RRC with MCP in Figure 3 are 0.88, 0.89, and 0.89. In addition, for class 1 in Figure 3, the *F*1 values are 0.88, 0.88, and 0.88, respectively. To explore the influence of MBPEP in dynamic ensemble models, optimal overproduction and learning performance are used as metrics in Figure 4. Different from Figure 3, META-MCP, KNORA-MCP, and RRC-MCP in Figure 4 are processed by Pareto optimization during the overproduction stage. META, KNORA, and RRC show that ensemble pruning model is learned by Pareto optimization but not MCP. From the top panel of Figure 4, it can be seen that META and KNORA-Eliminate models with MCP achieve superior learning performance to those without MCP. For RRC, RRC-MCP achieves comparable learning performance to that of RRC. From the bottom panel of Figure 4, it can be seen that META and RRC with MCP achieve an optimal overproduction pool size compared to that without MCP. For the KNORA-Eliminate model, KNORA-MCP achieves a comparable pool size to that without MCP.

### 3.4. Comparison

To measure the classification performance, test error and average optimized ensemble size are calculated using MBPEP and compared with the other ensemble methods. The results are shown in Tables 2 and 3. The test errors and optimal ensemble sizes of each model are not fixed for each experiment. For calculation with the benchmarked sets, each result is executed 30 times, and the average results are shown in Tables 2 and 3. The winners are bolded for MBPEP in Tables 2 and 3. The comparison methods are full bagging, RE pruning, Kappa pruning, CM pruning, MDM pruning, and EA pruning. RE pruning, Kappa pruning, CM pruning, and MDM pruning are dynamic ensemble pruning models that use competence ordering to estimate the competence of each learner; EA pruning is based on the optimized dynamic pruning model.

In Table 2, it can be seen that MBPEP achieves the lowest test error for 10 of 16 datasets (10/16). Meanwhile, the datasets with lowest test errors are full bagging, RE pruning, Kappa pruning, CM pruning, MDM pruning, and EA pruning, with 2, 3, 4, 3, 1, and 1 out of 16 datasets, respectively. Thus, MBPEP has been demonstrated to achieve better performance than the other methods. However, according to Demsar [36], it is inappropriate to use a single criterion to evaluate the performance of an ensemble classifier. In this section, the pairwise significance for numbers of direct wins is validated between MBPEP and the other algorithms using a sign test. Notably, a win is counted as 1 and a tie is counted as 0.5 for each dataset to compare MBPEP with the other algorithms in Tables 2 and 3. From Table 2, the numbers of direct wins are 13, 10.5, 11, 10, 11, and 11 when comparing MBPEP with the other methods.

We also measure the optimal ensemble size in Table 3. It can be easily found that the EA algorithm needs to query more sublearners during the aggregation process. This phenomenon has been explained by Zhou et al. [27]. According to Table 3, MBPEP achieves the lowest ensemble size on 81.25% (13/16) of the datasets, while the other methods achieve the lowest ensemble size in less than 19% (3/16). The results from Tables 2 and 3 support the claim that the performance of MBPEP is superior to those of the competence ordering method and the optimization-based pruning method for most datasets. The reason for this result is that MBPEP can simultaneously minimize classification error and ensemble size, whereas competence ordering ensemble methods (RE pruning, Kappa pruning, CM pruning, and MDM pruning) focus on optimizing only one objective, such as diversity, but neglect the others.

### 3.5. Robustness of Classification

The robustness of the algorithms is an important indicator for measuring classifier performance. It can reflect the fault tolerance of the algorithms. In this section, the ensemble classifiers are constructed under different levels of the Gaussian noise. The noise levels are determined using the variance intensity of the Gaussian noise. When the experiment is executed, the test errors of the ensemble classifiers are modified when the training sets are corrupted by 0.00, 0.02, 0.04, and 0.08 noise. The results are measured using the 7 datasets described in Table 4. The average test errors are shown in Table 4, and each one is calculated 50 times. To better evaluate the robustness of MBPEP, the performances of full bagging and the other ensemble pruning methods are also measured. The winners are bolded for MBPEP in Table 4.

In Table 4, it can be seen that the test errors of all methods increased with the intensity of the Gaussian noise. For example, MBPEP can achieve a 21.4% test error when there is no noise to corrupt the training set of the sonar dataset. However, the test error reaches 32.3% when the training set is corrupted by 0.08 Gaussian noise. According to Table 4, MBPEP achieves the lowest test error on 5, 5, 6, and 7 datasets of the 7 datasets under the different noise intensities. Specifically, when the variance intensity of the Gaussian noise is larger than 0.04, MBPEP performs better than the other methods. For example, the test error of MBPEP reaches 20.80% on the waveform dataset when there is no noise to corrupt the training set. It is not the best classification performance of all of the comparison methods, but when the variance of the noise is larger than 0.04, MBPEP achieves the lowest test errors. The number of winners for MBPEP increases with noise intensity, which demonstrates that MBPEP is robust with respect to Gaussian noise.

### 3.6. Application to the Pattern Recognition Task

**Figure 5:**
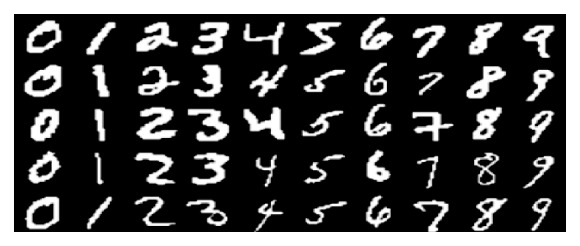
Examples of handwritten digits from the MNIST.

MBPEP is applied to handwritten digital character pattern recognition. Digital character recognition is an important research field in computer vision. MNIST [37] is a standard digital character benchmark dataset that contains 60000 and 10000 grayscale images for training and testing, respectively. Each image is mapped to 10 classes that include the digits 0 to 9.  Figure 5 shows samples of MNIST. To evaluate the generalization performance of the pruning ensemble method on MNIST, it was tested 30 times.

**Figure 6:**
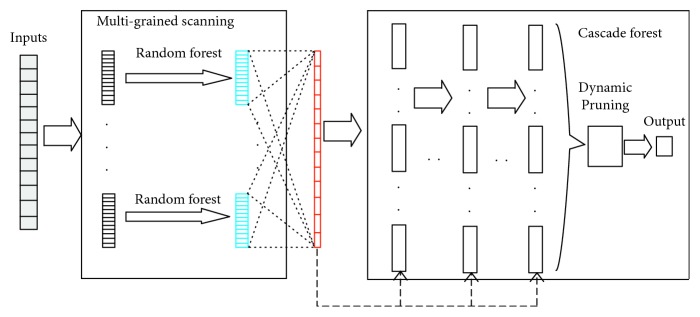
Modified version of gc-Forest.

In this section, MBPEP and the five dynamic pruning methods that were mentioned in Section 3.2 are applied to the deep forest framework multigrained cascade forest (gc-Forest) [1]. To validate the efficiency of MBPEP, the average max process has been replaced by dynamic pruning in gc-Forest. We call gc-Forest with the dynamic pruning process the modified version of gc-Forest. The two modules in gc-Forest, i.e., multigrained scanning and cascade forest, are reserved. The modified version of gc-Forest is shown in Figure 6.

**Figure 7:**
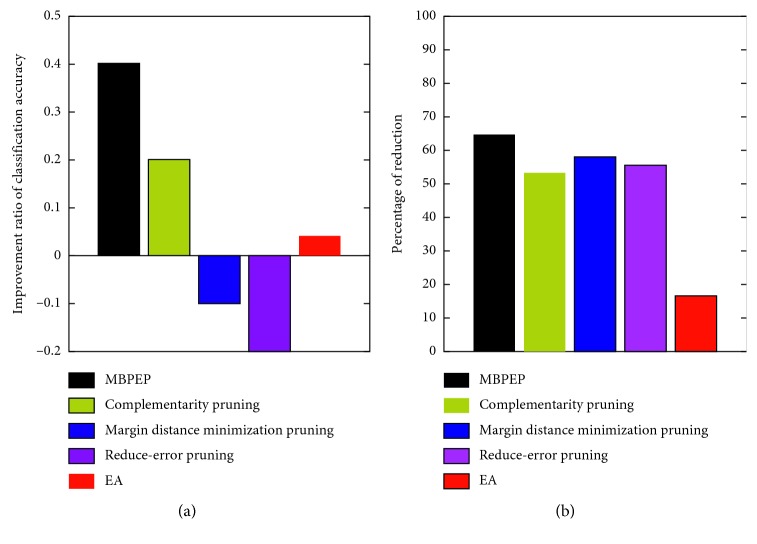
Performance of pruning ensemble algorithms with full bagging on digital character recognition. (a) Improvement ratio performance of the classification performance. (b) Reduction percentage of the ensemble size.

According to Figure 6, the overall learning procedure of the modified version of gc-Forest has the same construction as the original version of gc-Forest. The number of raw input features is scaled using the scanning method on the novel features (printed in blue blocks). The scaling process is determined using the scaling window sizes. The generated features are concatenated to the large feature vectors (printed in red blocks) that are prepared to enter the cascade forest. To encourage diversity and improve pruning efficiency for the construction of the cascade forest, each layer consists of 100 random forests in this section. The number of layers is self-adapted until the validation performance is below the error tolerance. We introduce a metric, namely, the improvement ratio of the test error, in this section. Specifically, the original version of gc-Forest is used as a baseline. The results are shown in Figure 7(a). In addition, the percentage of reduction for the number of optimized learners that has been compared with the original ensemble size is shown in Figure 7(b). The different colors in Figure 7 denote the different ensemble methods.

According to Figure 7(a), it can be seen that not all ensemble methods can improve the classification accuracy when compared with the original version of gc-Forest. The modified version of gc-Forest with MBPEP could improve the classification accuracy by 0.4% over that of the original version. Meanwhile, the other methods can improve the classification accuracy by 0.2%. From Figure 7(b), the results show that MBPEP can store fewer sublearners during aggregation. MBPEP reduces the query quantity by approximately 64.4% for the MBPEP algorithm when combining these sublearners into the final hypotheses. The other four ensemble methods need to query more sublearners, which increases the time consumption of the majority voting process. Thus, MBPEP has apparent advantages with less storage and higher computational efficiency than the other pruning methods.

## 4. Conclusions

The dynamic ensemble pruning technique is an important strategy for improving the performance of ensemble classifiers when a subset of ensemble pools is used as a classification expert with respect to the incoming set. During the overproduction stage of the dynamic ensemble pruning model, since selecting the optimal ensemble size is an NP-hard optimization problem, the initial ensemble pool size is fixed. Many previous publications mainly address the pruning criteria during the pruning stage, and few studies utilize the local margin information for learners to select the expert in an ensemble. In this paper, MBPEP is proposed.

First, the Pareto optimization algorithm is used by MBPEP to globally search for the feasible ensemble pool size during the overproduction stage. The “domination” computation between the learning performance and the ensemble pool size is continuously estimated by Pareto optimization. Second, the margin entropy for every learner in the ensembles is used to locally determine the expert that can detect the classes in the indecision region. Several experiments are conducted to demonstrate the advantages of MBPEP with respect to other state-of-art pruning methods (RE pruning, Kappa pruning, complementarity measure pruning, MDM pruning, RRC pruning, KNORA-Eliminate, META-DES, and EA ensemble method). Finally, MBPEP is applied to the deep forest framework to conduct handwritten digital character recognition tasks. Compared to the original version of gc-Forest, the average max process has been replaced by MBPEP-based gc-Forest. This modified version of gc-Forest with MBPEP can improve the classification accuracy while using fewer sublearners when combining the final hypotheses.

In the future, we would like to focus on merging ensemble methods with deep learning frameworks. The combination of ensemble methods and deep learning construction has become an advanced research direction in machine learning. For example, deep ensemble learning [2] has been demonstrated as a powerful tool for addressing various recognition tasks.

## Figures and Tables

**Algorithm 1:**
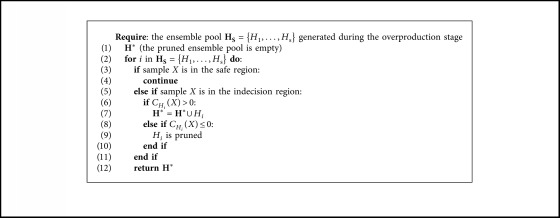
MCP algorithm.

**Table 1:** Benchmark datasets used in the experiments.

Data set	#Examples	#Attributes
Australian	690	14
Breast cancer	286	9
Liver	345	6
Heart-statlog	270	13
Waveform	1000	20
Ionosphere	351	34
Kr-vs-kp	3196	96
Letter	20,000	16
German	306	3
Diabetes	76	4
Sonar	208	60
Spambase	4601	57
Tic-tac-toe	958	9
Vehicle	846	18
Vote	435	16
Pima	768	8

**Table 2:** Test error comparison.

Data sets	Ours	Full bagging	RE pruning	Kappa pruning	CM pruning	MDM pruning	EA pruning
Australian	**11.62%**	14.30%	14.40%	14.30%	14.50%	14.80%	14.30%
Breast cancer	**23.83%**	27.90%	27.70%	28.70%	29.20%	29.50%	27.50%
Liver	**25.73%**	32.70%	32.00%	32.60%	30.60%	33.70%	31.70%
Heart-statlog	23.50%	19.50%	18.70%	20.10%	19.90%	19.90%	19.60%
Waveform	20.80%	22.20%	20.50%	20.90%	20.30%	20.10%	20.50%
Ionosphere	**5.70%**	9.20%	8.60%	8.40%	8.90%	10.00%	9.30%
Kr-vs-kp	**0.90%**	1.50%	1.00%	1.00%	1.10%	1.10%	1.20%
Letter	8.52%	5.90%	4.80%	4.80%	4.80%	5.70%	5.30%
German	**22.54%**	25.30%	24.10%	24.10%	23.90%	24.00%	24.30%
Diabetes	**22.16%**	25.10%	24.30%	24.30%	24.10%	24.20%	24.00%
Sonar	**21.40%**	26.60%	26.70%	24.90%	25.00%	26.80%	25.10%
Spambase	6.93%	6.80%	6.60%	6.60%	6.60%	6.80%	6.60%
Tic-tac-toe	15.67%	16.40%	13.50%	13.20%	13.20%	14.50%	13.80%
Vehicle	**21.97%**	22.80%	22.60%	23.30%	23.40%	24.40%	23.00%
Vote	4.40%	4.70%	4.40%	4.10%	4.30%	4.50%	4.50%
Optdigits	**3.20%**	3.80%	3.60%	3.50%	3.60%	3.70%	3.50%
Count of wins	**10**	2	3	4	3	1	1
Direct wins		13	10.5	11	10	11	11

**Table 3:** Average optimized ensemble size.

Data sets	Ours	RE pruning	Kappa pruning	CM pruning	MDM pruning	EA pruning
Australian	**7.2**	12.5	14.7	11.0	8.5	41.9
Breast-cancer	**7.2**	8.7	26.1	8.8	7.8	44.6
Liver	**7.4**	13.9	24.7	15.3	17.7	42
Heart-statlog	**9.6**	11.4	17.9	13.2	13.6	44.2
Waveform	**9**	12.3	15.6	11.7	11.5	42.4
Ionosphere	9.1	7.9	10.5	8.5	10.7	48.8
Kr-vs-kp	**4.3**	5.8	10.6	9.6	7.2	45.9
Letter	**12.5**	15.1	13.8	12.9	23.2	38.3
German	**7.0**	9.4	13.6	11.5	19.7	41.2
Diabetes	**11.7**	14.3	16.8	12.6	18.1	42.8
Sonar	13.2	11.0	20.6	13.9	20.6	43.1
Spambase	**12.6**	18.5	20.0	19.0	28.8	39.7
Tic-tac-toe	**13.0**	16.1	17.4	15.4	28.0	39.8
Vehicle	**7.0**	15.7	16.5	11.2	21.6	41.9
Vote	3.5	3.2	5.1	5.4	6.0	47.8
Optdigits	**22.3**	25.0	25.2	21.4	46.8	41.4
Count of wins	**13**	3	0	0	0	0
Direct wins		13	16	14	16	16

**Table 4:** Test errors in the noisy classification problem.

Noise	Data sets	Ours (%)	Full bagging (%)	RE pruning (%)	Kappa pruning (%)	CM pruning (%)	MDM pruning (%)	EA pruning (%)
0.00	Diabetes	**22.10**	25.10	24.30	24.30	24.30	24.20	24.00
	German	**22.50**	25.30	24.10	24.10	23.90	24.00	24.30
	Heart-statlog	23.50	19.50	18.70	20.10	19.90	19.90	19.60
	Ionosphere	**5.70**	9.20	8.60	8.40	8.90	10.00	9.30
	Sonar	**21.40**	26.60	26.70	24.90	25.00	26.80	25.10
	Vehicle	**21.90**	22.80	22.60	23.30	23.40	24.40	23.00
	Waveform	20.80	22.20	20.50	20.90	20.30	20.10	20.50

0.02	Diabetes	**24.20**	26.60	26.60	26.30	26.70	26.40	27.00
	German	**24.40**	27.70	27.70	28.10	27.00	27.10	27.60
	Heart-statlog	24.30	23.20	22.70	21.60	22.00	22.20	23.10
	Ionosphere	**7.20**	13.10	11.80	13.00	11.50	11.30	12.20
	Sonar	**23.40**	28.10	24.00	26.20	24.60	23.50	25.30
	Vehicle	**25.80**	33.00	29.90	29.90	29.60	30.10	29.80
	Waveform	25.20	26.80	24.30	25.80	24.20	23.90	24.50

0.04	Diabetes	**27.30**	30.10	29.60	29.70	29.60	29.40	30.10
	German	**29.10**	31.00	30.10	31.30	29.90	29.70	30.70
	Heart-statlog	28.00	28.20	28.10	26.50	27.50	27.50	28.00
	Ionosphere	**11.40**	17.70	15.80	18.30	15.70	15.90	17.50
	Sonar	**25.10**	30.10	28.10	29.60	27.70	25.90	28.30
	Vehicle	**25.10**	30.10	28.10	29.60	27.70	25.90	28.30
	Waveform	**27.50**	30.10	28.10	29.20	27.90	27.60	28.60

0.08	Diabetes	**27.50**	30.10	28.10	29.20	27.90	27.60	28.60
	German	**33.60**	36.20	36.10	36.40	35.90	36.10	38.20
	Heart-statlog	**32.40**	33.40	32.60	32.90	33.20	33.30	34.40
	Ionosphere	**26.40**	27.50	26.50	27.60	26.60	26.50	27.90
	Sonar	**32.30**	36.10	36.50	38.40	35.70	36.40	36.50
	Vehicle	**38.70**	43.00	40.60	42.00	40.50	40.20	41.60
	Waveform	**34.30**	37.60	35.80	36.80	35.60	35.30	37.40

## Data Availability

The datasets generated during and analysed during the current study are available in the UCI repository (https://archive.ics.uci.edu/ml/index.php) and the MNIST repository (http://yann.lecun.com/exdb/mnist/).
